# Chinese expert consensus on the choice of initial cholesterol-lowering strategy

**DOI:** 10.3389/fphar.2026.1798787

**Published:** 2026-05-19

**Authors:** Na-Qiong Wu, Xiao Wang, Ping Ye, Hong Chen, Zhen-Yue Chen, Dong Zhao, Dao-Quan Peng, Yuan-Lin Guo, Ying Gao, Jin-Gang Yang, Cheng-Gang Zhu, Ke-Fei Dou, Jian-Jun Li, Cardiovascular Metabolic Medicine Task Group, National Cardiovascular Disease Expert Committee, Hong Chen, Zhen-Yue Chen, Xiang Cheng, Ke-Fei Dou, Ying Gao, Yuan-Lin Guo, Kai Huang, Jian-Jun Li, Li-Wen Li, Yong Li, Chun Liang, Xiao Wang, Dao-Quan Peng, Na-Qiong Wu, Ping Ye, Jin-Gang Yang, Da-Qing Zhang, Dong Zhao, Jia-Jun Zhao, Zhi-Guang Zhou, Cheng-Gang Zhu

**Affiliations:** 1 Cardiometabolic Center, National Center for Cardiovascular Diseases, Fuwai Hospital, Chinese Academy of Medical Sciences and Peking Union Medical College, Beijing, China; 2 Department of Geriatric Cardiology, National Clinical Research Centre for Geriatric Disease, Chinese PLA General Hospital, Beijing, China; 3 Department of Cardiology, Beijing Key Laboratory of Early Prediction and Intervention of Acute Myocardial Infarction, Center for Cardiovascular Translational Research, Peking University People’s Hospital, Beijing, China; 4 Department of Cardiology, Ruijin Hospital, Shanghai Jiao Tong University School of Medicine, Shanghai, China; 5 Department of Epidemiology, Beijing Anzhen Hospital, Capital Medical University, Beijing Institute of Heart, Lung, and Blood Vessel Diseases, Beijing, China; 6 Department of Cardiology, The Second Xiangya Hospital, Central South University, Changsha, China

**Keywords:** atherosclerotic cardiovascular disease, Chinese, cholesterol-lowering therapy, expert consensus, initial

## Abstract

It has been confirmed that cholesterol-lowering therapy, particularly the reduction of low-density lipoprotein cholesterol (LDL-C) levels, represents the most effective measure for decreasing the incidence of atherosclerotic cardiovascular disease and related cardiovascular events. Previous studies have shown that active reduction of cholesterol levels significantly increases the goal attainment rate of LDL-C and improves clinical outcomes. Although many cholesterol-lowering medications are currently available in clinical practice and the cholesterol lowering treatment status has improved, the real-world cholesterol management in China, especially the goal attainment rate of LDL-C, is not satisfactory. This issue is closely related to the selection of initial cholesterol-lowering treatment strategies. Furthermore, clinical research has demonstrated that the safety and adherence of cholesterol-lowering therapy are also critical factors influencing treatment effectiveness. In response, the National Cardiovascular Disease Expert Committee Cardiovascular Metabolic Medicine Professional Committee has organized domestic experts to systematically and comprehensively review methods and scientific choices for initiating cholesterol-lowering therapy in the Chinese population and developed a Chinese expert consensus on the selection of initial cholesterol-lowering strategies based on current evidence. It aims to propose optimized initial treatment regimens tailored to individual characteristics, thereby further improving the prevention and management of dyslipidemia in Chinese population.

## 1 Introduction

Cardiovascular disease (CVD) is a leading cause of mortality worldwide. Global Burden of Disease (GBD) data show that an estimated 19.2 million people died from CVD in 2023, accounting for one-third of all deaths worldwide (Global Burden of Cardiovascular Diseases and Risks 2023 Collaborators, 2025). Notably, CVD is also the leading cause of death among both urban and rural residents in China, responsible for about two out of every five deaths (National Center For Cardiovascular Diseases, 2025). Among them, atherosclerotic cardiovascular disease (ASCVD), primarily comprising ischemic stroke and ischemic heart disease, constitutes roughly 63% of CVD mortality in the Chinese population (Global Burden of Cardiovascular Diseases and Risks 2023 Collaborators, 2025). Studies in primary and secondary prevention have consistently demonstrated that cholesterol-lowering therapy significantly reduces the incidence of ASCVD and the risk of subsequent cardiovascular events (Ference et al., 2017). LDL-C is a “pathogenic” risk factor for ASCVD, and it is recognized as a primary intervention target in both domestic and international guidelines.

**FIGURE 1:**
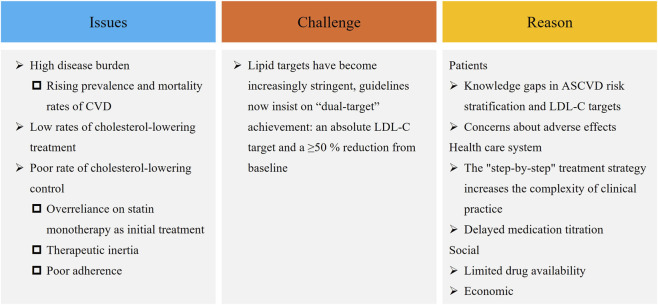
Challenges and current issues in cholesterol management for the Chinese population. CVD, cardiovascular disease; LDL-C, low-density lipoprotein cholesterol; ASCVD, atherosclerotic cardiovascular disease.

The cholesterol-lowering treatment strategies discussed in this consensus primarily refer to therapeutic approaches aimed at reducing LDL-C levels. Currently, most domestic and international guidelines generally adopt a “stepwise” approach in cholesterol-lowering therapy, that is, statins as the starting treatment, and if target levels are not achieved, the sequential addition of cholesterol absorption inhibitors and/or proprotein convertase subtilisin/kexin type 9 inhibitors (PCSK9i) is recommended. European and American guidelines particularly emphasize initiating high-intensity/maximum tolerated dose of statin therapy as initial treatment strategy, which was initially proposed in an era when statins were the only option and the LDL-C target for secondary prevention was <1.8 mmol/L. In recent years, as LDL-C targets have become increasingly stringent, this conventional stepwise strategy inevitably increases the difficulty in achieving LDL-C targets, reduces of treatment adherence due to adverse effects, and affects the operability in real-world clinical practice (Figure 1) (Li et al., 2023; Rao et al., 2025; Vrints et al., 2024). Meanwhile, accumulating evidence confirms that early and intensive cholesterol-lowering enables patients to reach LDL-C targets sooner, resulting in more substantial cardiovascular benefits. In response, the National Cardiovascular Disease Expert Committee Cardiovascular Metabolic Medicine Professional Committee developed this consensus document. The expert panel formulated the consensus based on a comprehensive literature review, employing a combination of the consensus development conference and Delphi methods. Initially, a consensus literature database was established by retrieving relevant studies on initial cholesterol-lowering strategies from PubMed, Embase, and https://clinicaltrials.gov. Subsequently, the panel thoroughly deliberated on the gathered evidence and clinical issues to draft preliminary recommendations, which were then subjected to formal voting. Consensus was defined as an agreement threshold of >75% among the experts, thereby establishing the final expert opinions. It aims to provide clinicians with a simple and practical framework for initiating cholesterol-lowering therapy, ultimately improving cholesterol management in the Chinese population, enhancing LDL-C goal attainment rates, and advancing cardiovascular disease prevention and treatment outcomes.

## 2 Current status of lipid management in China

China faces a heavy burden of dyslipidemia, characterized by a high prevalence but low rates of treatment and control, leaving the overall status of lipid management far from optimistic. Preliminary findings from the “Cardiovascular Diseases and Risk Factors Surveillance among Chinese Residents” project (2020–2022) show that among Chinese adults aged ≥18 years, the prevalence, awareness, treatment, and control rates of dyslipidemia were 38.1%, 11.7%, 10.1%, and 4.8%, respectively (National Center For Cardiovascular Diseases, 2025). China HAERT showed that in high- ASCVD risk populations, the rate of LDL-C ≤2.6 mmol/L was 42.9%, while in patients with very high ASCVD risk, the rate of LDL-C ≤1.8 mmol/L was 26.6%, the treatment rate was 14.1%, and the LDL-C goal attainment rate in treatment patients was 44.8% (Lu et al., 2021).

Insufficient intensity of cholesterol-lowering therapy is a major reason for the low LDL-C goal attainment rate. The Dyslipidemia International Study-China (DYSIS-China) showed that among Chinese patients receiving lipid-lowering therapy for at least 3 months, statin monotherapy remained the dominant treatment, and the proportion of combination therapy was only about 2% (Zhao S. et al., 2014). Furthermore, poor patient adherence and clinical inertia also contribute to the challenges in lipid management, reducing goal attainment rates and increasing cardiovascular risk (Khunti et al., 2018; Raebel et al., 2013). Therefore, it is necessary to develop more effective cholesterol-lowering treatment strategies to further improve LDL-C goal attainment rates and reduce the burden of cardiovascular disease.

## 3 The importance of the initial cholesterol-lowering strategy

**FIGURE 2:**
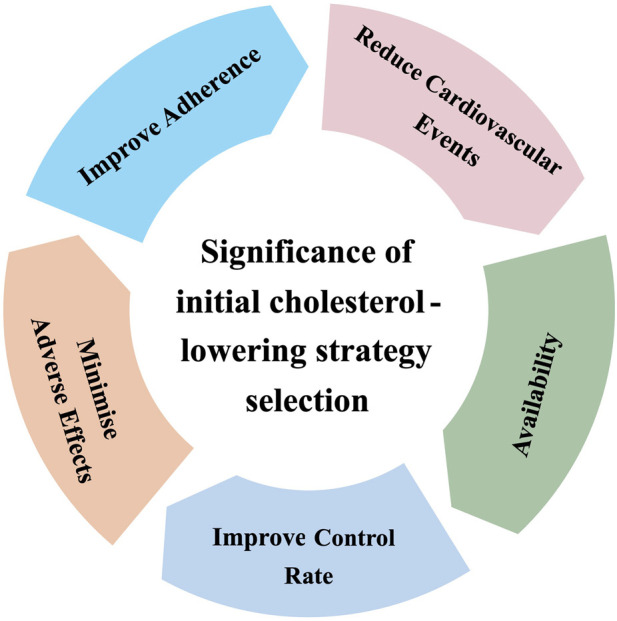
Significance of initial cholesterol-lowering strategy selection.

Based on ASCVD risk stratification and the corresponding LDL-C targets, it is imperative to adopt an individualized strategy for managing cholesterol. As recent national and international guidelines have progressively lowered LDL-C goals, the proportion of patients achieving target levels on statin monotherapy has further declined (Ray et al., 2021). Consequently, a greater number of patients require regimen adjustments to achieve the targets specified by the guidelines. Multiple real-world studies have demonstrated that early, intensive cholesterol-lowering therapy can improve the LDL-C goal attainment rate and reduce the risk of major adverse cardiovascular events (MACE) (Patti et al., 2025; Schubert et al., 2024). This suggests that a rational initial cholesterol-lowering treatment plan can help streamline the treatment pathway and mitigate clinical inertia, thereby enhancing patient adherence and goal attainment rates. Furthermore, due to variations in adverse drug reactions across different populations and differences in drug availability among healthcare institutions, clinical decision-making must also comprehensively consider patient tolerance and accessibility of medications. Therefore, the selection of an initial cholesterol-lowering strategy is not a simple matter of drug choice, but rather requires a balanced consideration of multiple factors, including efficacy, adherence, safety, and practicality (Figure 2). The appropriate initial cholesterol-lowering regimen should be selected based on ASCVD risk stratification and individual patient characteristics to achieve early, sustained, and safe attainment of cholesterol targets, thereby effectively reducing cardiovascular risk.

EXPERT OPINION 1

1. The choice of initial cholesterol-lowering therapy is an important strategy to improve early LDL-C target attainment rates and patient adherence.

## 4 ASCVD risk stratification in the Chinese guideline for lipid management

The core of the 2023 Chinese Guidelines for Lipid Management in ASCVD risk stratification is to assess and identify the risk of ASCVD over the next 10 years and even throughout the patient’s lifetime, thereby guiding intervention intensity based on the estimated risk level. The risk stratification adopts a multi-level system ranging from low risk to ultra-high risk, and sets cholesterol-lowering treatment targets based on different risk categories. Detailed risk stratification criteria and cholesterol-lowering treatment targets are provided in Table 1 (Li et al., 2023).

**TABLE 1:** Risk stratification and cholesterol-lowering targets for ASCVD in Chinese adults (Li et al., 2023).

Cardiovascular risk categories in Chinese adults	LDL-C recommended target value
Ultra-high risk: ≥2 severe ASCVD events (e.g., myocardial infarction, ischemic stroke, peripheral arterial revascularization, or amputation), or 1 severe ASCVD event combined with ≥2 high-risk factors FH with ASCVD	<1.4 mmol/L and >50% reduction from baseline
Very high risk: Other ASCVD patients who do not meet the ultra-high risk criteria Diabetic patients (≥40 years) FH with subclinical ASCVD	<1.8 mmol/L and >50% reduction from baseline
Medium and high risk: Diabetic patients (<40 years) LDL-C ≥ 4.9 mmol/L CKD (stage 3–4) FH Hypertension with ≥1 additional risk factor^a^ Carotid plaque without any other risk factors^a^ Risk factors ≥3^a^	<2.6 mmol/L^b^
Low risk Individuals not meeting the above criteria	<3.4 mmol/L

LDL-C, low-density lipoprotein cholesterol; ASCVD, atherosclerotic cardiovascular disease; HDL-C, high-density lipoprotein cholesterol; CKD, chronic kidney disease; BMI, body mass index. FH, familial hypercholesterolemia.

^a^ Risk factors included age >45,55 years (male/female); smoking, HDL-C <1.0 mmol/L, BMI ≥28 kg/m2; family history of premature ASCVD (male <55 years, female <65 years).

^b^ For individuals with ≥2 clinical conditions, the target value should be adjusted downward to <1.8 mmol/L.

## 5 Initial cholesterol-lowering therapy: regimens, evidence, and recommended populations

Strategies for cholesterol-lowering treatment include lifestyle interventions and pharmacotherapy. Lifestyle interventions are the foundation of cholesterol-lowering treatment. When lifestyle interventions fail to achieve lipid lowering goals, the addition of lipid-lowering drugs should be considered. In clinical practice, a patient’s ASCVD risk stratification and baseline LDL-C level determine the LDL-C target and the required reduction, which are the key determinants of the initial lipid-lowering strategy. Furthermore, comorbidities, potential drug–drug interactions, and patient preferences should also be comprehensively considered to ensure the development of an individualized and sustainable treatment plan.

### 5.1 Cholesterol-lowering monotherapy regimen

#### 5.1.1 High-intensity/maximum tolerated dose of statins

Statins are recognized as the cornerstone of cholesterol-lowering therapy. The mechanism of action is competitive inhibition of 3-hydroxy-3-methylglutaryl-coenzyme A (HMG-CoA) reductase, the rate-limiting enzyme of the mevalonate pathway, thereby reducing cholesterol synthesis. Concurrently, statins upregulate LDL receptors (LDLR), leading to a reduction in cholesterol levels. Based on treatment intensity, statins can be categorized into two main classes: high-intensity and moderate-intensity. High-intensity statins refer to daily treatment doses that can lower LDL-C by ≥50%, which includes atorvastatin 40–80 mg/day and rosuvastatin 20–40 mg/day. The maximum tolerated statin dose is defined as the highest dose that provides the greatest cholesterol-lowering effect while remaining tolerable for the patient.

Studies have demonstrated that the clinical benefit of statins in reducing ASCVD events exhibits a linear correlation with the magnitude of LDL-C reduction. The Cholesterol Treatment Trialists’ (CTT) meta-analysis showed that each 1 mmol/L reduction in LDL-C was associated with a 22% relative risk reduction in major vascular events within 5 years (Baigent et al., 2010). Trials that initiated cholesterol-lowering therapy with high-intensity statins were predominantly conducted in Western populations (see Supplementary Table S1). Both the Myocardial Ischemia Reduction with Aggressive Cholesterol-lowering (MIRACL) and the Pravastatin or Atorvastatin Evaluation and Infection Therapy-Thrombolysis in Myocardial Infarction 22 (PROVE IT-TIMI 22) trials demonstrated that initiating high-intensity atorvastatin (80 mg/day) in hospitalized patients with acute coronary syndrome (ACS) resulted in significantly greater reductions in LDL-C levels and lower risk of primary endpoint events compared to either placebo or moderate-intensity statin therapy (Rouleau, 2005; Schwartz et al., 2001). The Treating to New Targets (TNT) study showed that in patients with stable coronary artery disease, randomization to atorvastatin 80 mg/day reduced the risk of primary composite cardiovascular events by 22% compared to atorvastatin 10 mg/day (LaRosa et al., 2005). The Stroke Prevention by Aggressive Reduction in Cholesterol Levels (SPARCL) trial showed that a reduction of 16% of stroke recurrence risk in patients with ischemic stroke or transient ischemic attack (TIA) who received atorvastatin 80 mg daily (Amarenco et al., 2006).

However, the clinical utility of statins is limited by the “6% rule”, whereby doubling the statin dose yields only an approximately 6% additional reduction in LDL-C levels (Li et al., 2023). On the other hand, statin-associated adverse effects increase substantially with higher doses, which may lead to reduced treatment adherence (Bates et al., 2009). Furthermore, Chinese patients exhibit poor tolerance to high-dose statin therapy. Consequently, the strategy of initiating lipid-lowering therapy with high-intensity or the maximum tolerated dose of statin is applicable to high and very-high-risk Western populations with ASCVD, such as those with stable coronary heart disease, ACS, and ischemic stroke/TIA. This approach is endorsed by numerous international guidelines, which recommend starting with high intensity/maximum tolerated dose of statin therapy. If treatment goals are not achieved, the addition of a cholesterol absorption inhibitor (e.g., ezetimibe) is advised, followed by a PCSK9i if targets remain unmet (Byrne et al., 2023; Mach et al., 2020; Rao et al., 2025; Visseren et al., 2021; Vrints et al., 2024). In principle, this strategy is not recommended as the initial lipid-lowering regimen for Chinese populations.

#### 5.1.2 Moderate-intensity statins

2023 Chinese Guidelines for Lipid Management defined moderate-intensity statins as those that reduce LDL-C by 25%–50% at daily therapeutic doses. The different types and doses of moderate-intensity statins are detailed in Table 2 (Li et al., 2023).

**TABLE 2:** Moderate-intensity statins.

Cholesterol-lowering intensity	Agents and dosages
Moderate-intensity statins (25%–50% reduction in LDL-C at daily dose)	Atorvastatin 10–20 mg Rosuvastatin 5–10 mg Fluvastatin 80 mg Lovastatin 40 mg Pitavastatin 1–4 mg Pravastatin 40 mg Simvastatin 20–40 mg Xuezhikang 1.2 g

LDL-C, low-density lipoprotein cholesterol.

The clinical evidence for initiating cholesterol-lowering therapy with moderate-intensity statins is detailed in Supplementary Table S2. The West of Scotland Coronary Prevention Study (WOSCOPS) demonstrated that pravastatin (40 mg/day) reduced LDL-C in patients with hypercholesterolemia by 26%, significantly lowering the risk of coronary heart disease events by 31% and cardiovascular mortality by 32% compared with placebo (Shepherd et al., 1995). A large-scale randomized controlled trial (RCT) demonstrated that in patients with CVD or other high-risk conditions, simvastatin 40 mg/day significantly reduced the risk of new-onset stroke by 25% and major vascular events by 20% compared to placebo (Collins et al., 2004). Notably, the pharmacokinetics of statins differ between Asian and Caucasian populations (Li et al., 2024), Lower doses of statins produce LDL-C reductions and cardiovascular benefits similar to those required by higher doses in non-Asians (Giraldez et al., 2008; Li et al., 2025; Tomlinson et al., 2018; Zhao S. P. et al., 2014). The China Intensive Lipid Lowering with Statins in Acute Coronary Syndrome (CHILLAS) study compared the impact of moderate-dose statin therapy (atorvastatin 10 mg/day or equivalent) *versus* double-dose statin therapy (atorvastatin 20–40 mg/day or equivalent) on major adverse cardiovascular events (MACE) in ACS patients over a 2-year treatment period. Results showed the moderate-dose group reduced LDL-C by 20.2%, while the double-dose group reduced LDL-C by 26.6%. There was no statistically significant difference in the incidence of primary endpoint events between the two groups (Lu et al., 2008). The China Coronary Heart Disease Secondary Prevention Study (CCSPS) showed that in Chinese post-myocardial infarction patients, Xuezhikang significantly reduced LDL-C by 17.6% compared to placebo, reduced the relative risk of major coronary events by 45%, and all-cause mortality by 33%, with no significant difference in overall adverse events compared to placebo (Lu et al., 2008).

Although moderate-intensity statin enables most Chinese patients at low to moderate cardiovascular risk to achieve LDL-C targets, its cholesterol-lowering efficacy remains limited and is often insufficient for high-risk, very high-risk, and ultra-high ASCVD risk patients to reach target. The DYSIS-China study (n = 25,697) revealed that when moderate-intensity statins were used in high-risk and very high-risk patients, the LDL-C goal attainment rate was only 39.7% (Zhao S. et al., 2014).

Therefore, this consensus recommends moderate-intensity statins as the initial cholesterol-lowering strategy for patients in the primary prevention of ASCVD, based on their baseline cholesterol levels. Xuezhikang, a moderate-intensity natural lipid-lowering agent containing naturally derived lovastatin, may be considered as an alternative initial cholesterol-lowering strategy for statin intolerant patients.

EXPERT OPINION 2

1. High-intensity or maximum tolerated dose of statin therapy is the recommended initial cholesterol-lowering strategy in foreign guidelines.
2. Moderate-intensity statins are the cornerstone of the initial cholesterol-lowering strategy and combination therapy recommended by Chinese guidelines.

#### 5.1.3 Cholesterol absorption inhibitors

Cholesterol absorption inhibitors are a class of lipid-lowering agents that reduce serum cholesterol levels by inhibiting the intestinal absorption of cholesterol. There are two main types currently available: ezetimibe and hybutimibe.

Previous studies have shown that ezetimibe monotherapy can reduce LDL-C by 18%–20% (Pandor et al., 2009), while hybutimibe monotherapy can achieve an LDL-C reduction of 10%–15% (Jiyan et al., 2022; Siyu et al., 2023). Ezetimibe Lipid-Lowering Trial on Prevention of Atherosclerotic Cardiovascular Disease in 75 or older (EWTOPIA 75) study demonstrated that in patients aged ≥75 years without coronary heart disease, treatment with ezetimibe 10 mg daily reduced LDL-C by 25.9% and lowered the risk of the primary composite endpoint by 34%. This supports ezetimibe as an initial lipid-lowering strategy for the primary prevention of ASCVD in the elderly (Ouchi et al., 2019).

Cholesterol absorption inhibitors are well tolerated with mild adverse events and are generally transient. Meta-analyses showed that the incidence of adverse events such as gastrointestinal adverse effects, musculoskeletal disorders, new onset diabetes, and elevated liver-enzyme with ezetimibe monotherapy was similar to placebo (Pandor et al., 2009; Wang et al., 2022). On the basis of available evidence, the present consensus recommends cholesterol absorption inhibitors as initial lipid-lowering therapy for primary prevention in population aged ≥75 years. It may also be considered as initial therapy in patients with statin intolerance (Li et al., 2023; Mach et al., 2020; Visseren et al., 2021).

EXPERT OPINION 3

1. Cholesterol absorption inhibitors are recommended as an initial cholesterol-lowering strategy for the primary prevention of ASCVD in individuals aged ≥75 years.
2. For patients with statin intolerance, initiation of a cholesterol absorption inhibitor may also be considered as a therapeutic option.

#### 5.1.4 Proprotein convertase subtilisin/kexin type 9 inhibitors (PCSK9i)

PCSK9i are a class of cholesterol-lowering agents that target PCSK9, which can reduce cholesterol levels by binding to PCSK9 or by inhibiting the synthesis of PCSK9 protein in hepatocytes, thereby preventing PCSK9-mediated low-density lipoprotein receptor (LDLR) degradation. Currently, PCSK9i available in China primarily include PCSK9 monoclonal antibodies and inclisiran.

Evidence supporting the use of PCSK9i as initial cholesterol-lowering therapy is primarily derived from primary prevention studies. Studies have demonstrated that initiating PCSK9i monotherapy can reduce LDL-C by 45%–57%, with a favorable safety profile. Studies have demonstrated that evolocumab monotherapy reduced LDL-C by 55%–57% in patients with hypercholesterolemia (Koren et al., 2014). Recaticimab monotherapy reduced LDL-C by 45%–49.6% (Xu et al., 2024). The Efficacy and Safety of Inclisiran as Monotherapy in Patients with Primary Hypercholesterolemia Not Receiving Lipid-Lowering Therapy (VICTORION-Mono) showed that inclisiran monotherapy reduced LDL-C by 46.5% among patients without previous ASCVD, diabetes, or familial hypercholesterolemia (FH) in primary prevention (Taub et al., 2025).

However, the current evidence supporting PCSK9i as initial monotherapy remains relatively limited. These studies have been conducted in patients with low-to-moderate ASCVD risk, and the outcome measure is limited to cholesterol reduction, with no established evidence for cardiovascular event benefit. Furthermore, except for recaticimab, other PCSK9i have not yet been approved for monotherapy indication in China. The long-term application of PCSK9i may also be constrained by issues related to adherence and cost-effectiveness. A real-world study in China revealed poor adherence to PCSK9i therapy, with a Proportion of Days Covered (PDC) of only 0.09 over 12 months. The discontinuation rate was 81.4% at 3 months and as high as 91.1% at 12 months, indicating that the majority of patients received only a single injection (Ye et al., 2025). Considering patient affordability, the cost of PCSK9 inhibitors is higher compared to statins and ezetimibe, and PCSK9i monotherapy has lower cost-effectiveness for primary or secondary prevention of ASCVD (Michaeli et al., 2023). Therefore, this consensus does not currently recommend the routine use of PCSK9i monotherapy as an initial lipid-lowering strategy in either primary or secondary prevention. Nevertheless, for patients who are statin-intolerant, or who still have concerns about adverse effects of statins after guideline-based doctor-patient communication, initiating therapy with PCSK9i monotherapy or in combination with a cholesterol absorption inhibitor is reasonable.

### 5.2 Cholesterol-lowering combination therapy regimen

#### 5.2.1 High-intensity/maximum tolerated statins combined with cholesterol absorption inhibitors

High-intensity or maximum tolerated statin combined with a cholesterol absorption inhibitor is also one of the initial cholesterol-lowering strategies. This combination leverages the complementary mechanisms of these agents, simultaneously inhibiting cholesterol synthesis and absorption.

High-intensity or maximum tolerated statin combined with a cholesterol absorption inhibitor can achieve an LDL-C reduction of approximately 60%–65%. In one randomized controlled trial, patients with hypercholesterolemia were assigned to receive either various doses of atorvastatin monotherapy (10–80 mg/day) or a combination therapy with ezetimibe. All combination therapy groups exhibited LDL-C reductions from baseline exceeding 50%, with an LDL-C attainment rate of 85%, both of which were significantly higher than the monotherapy groups. Specifically, the two groups receiving atorvastatin 40 mg/day and 80 mg/day combined with ezetimibe achieved LDL-C reductions of 56% and 61%, respectively (Ballantyne et al., 2003). Furthermore, a meta-analysis showed that the early addition of ezetimibe to high-intensity statin therapy significantly lowered LDL-C levels at follow-up time points of 7 days, 1 month, 3 months, and 1 year, and was associated with a significant reduction in the risk of recurrent cardiovascular events (Mahajan et al., 2024).

The 2025 Focused Update of the 2019 ESC/EAS Guidelines for the management of dyslipidaemias recommend initiating high-intensity statin combined with ezetimibe during hospitalization in treatment-naïve ACS patients to further reduce LDL-C levels (Mach et al., 2025). However, considering that the evidence for high-intensity statins in Chinese populations is limited and their tolerability is inferior to that observed in Western populations, this initial combination therapy is primarily recommended for Western patients with high and very high ASCVD risk, particularly those with ACS. For patients with FH who fail to achieve LDL-C targets with moderate-intensity statins combined with a cholesterol absorption inhibitor, initiating high-intensity statins combined with a cholesterol absorption inhibitor is also reasonable.

#### 5.2.2 Moderate-intensity statins combined with cholesterol absorption inhibitors

Moderate-intensity statin combined with a cholesterol absorption inhibitor (such as ezetimibe) is considered an optimal initial lipid-lowering strategy for early achievement of LDL-C targets in Chinese populations (Cannon et al., 2015a; Jarcho and Keaney, 2015; Li et al., 2025). Substantial evidence indicates that this combination regimen demonstrates superior efficacy and safety compared to high-intensity statin monotherapy (refer to Supplementary Table S3 for details).

In patients with hypercholesterolemia, moderate-intensity statin combined with ezetimibe can reduce LDL-C by 50%–60% from baseline, achieving a comparable lipid-lowering effect to that of atorvastatin 80 mg/day (54%) (Ballantyne et al., 2003; Li et al., 2023; Yang et al., 2017). Hybutimibe combined with atorvastatin 10 or 20 mg/day reduced LDL-C by 39% and 40% from baseline, respectively (Siyu et al., 2023). A meta-analysis including 47 RCTs showed statin combined with ezetimibe significantly reduced LDL-C and increased LDL-C attainment rate compared with double-dose statin (Mahmoud et al., 2025). Beyond lowering LDL-C, compared with statin monotherapy, moderate-intensity statin combined with ezetimibe therapy can further reduce atherosclerotic plaque burden. In patients with stable coronary artery disease undergoing elective percutaneous coronary intervention (PCI), atorvastatin 10–20 mg combined with ezetimibe significantly reduced plaque volume (Ueda et al., 2017). Additionally, moderate-intensity statin combined with ezetimibe therapy can further improve cardiovascular outcomes. Improved Reduction of Outcomes: Vytorin Efficacy International Trial (IMPROVE-IT) demonstrated that, compared with simvastatin monotherapy, simvastatin combined with ezetimibe further reduced LDL-C levels in hospitalized ACS patients, and the risk of the 7-year primary composite endpoint was reduced by 6.4% (Cannon et al., 2015a). Study of Heart and Renal Protection (SHARP) demonstrated that in patients with chronic kidney disease (CKD), moderate-intensity simvastatin combined with ezetimibe reduced the risk of primary atherosclerotic events by 17% compared to placebo (Baigent et al., 2011). In RCTs, the cardiovascular benefits of moderate-intensity statin combined with ezetimibe appear to be comparable with those of double-dose statin (Mahmoud et al., 2025). The RACING trial was the first large-scale RCT in an Asian population comparing moderate-intensity statin combined with ezetimibe with double-dose statin for ASCVD event prevention. Results demonstrated that the risk of primary composite endpoint events in the combination group was non-inferior to the high-intensity statin group (9.9% vs. 9.1%), with significantly higher rates of achieving LDL-C <1.8 mmol/L at years 1, 2, and 3 of treatment (Kim et al., 2022). But in real world, two Korean cohort studies showed that treatment with moderate-intensity statins combined with ezetimibe significantly reduced the risk of primary cardiovascular events compared to high-intensity statins in patients after PCI and with diabetes (Lee et al., 2025; Sohn et al., 2025).

**FIGURE 3:**
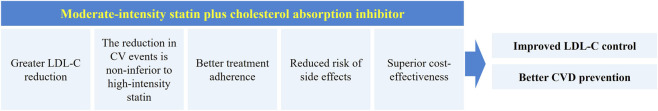
Advantages of moderate-intensity statin combined with cholesterol absorption inhibitor in Chinese population (Li et al., 2025). LDL-C, low-density lipoprotein cholesterol; CVD, cardiovascular disease.

Compared to high-intensity statin therapy, moderate-intensity statin combined with ezetimibe is associated with lower rates of adverse events such as liver function dysfunction, new-onset diabetes, and myalgia, as well as higher treatment adherence (Sydhom et al., 2024). Studies including RACING trial have shown that intolerance-related discontinuation or dose reduction is significantly less frequent with combination therapy than with high-intensity statin monotherapy (Kim et al., 2022; Lee et al., 2023). Therefore, for patients with ASCVD, those in primary prevention of ASCVD with baseline LDL-C ≥4.9 mmol/L or with FH, as well as high-risk diabetes or severe chronic kidney disease, this consensus recommends moderate-intensity statin combined with cholesterol absorption inhibitor (e.g., ezetimibe) as a preferred initial lipid-lowering strategy (Figure 3). It is noteworthy that real-world studies indicate that the use of a fixed-dose combination (FDC) of moderate-intensity statin and a cholesterol absorption inhibitor achieve higher LDL-C goal attainment rates and better safety profiles compared to free combinations of the individual agents (Samnaliev et al., 2025; Zambon et al., 2024).

EXPERT OPINION 4

1. The combination of moderate-intensity statins with cholesterol absorption inhibitors can significantly reduce LDL-C levels by more than 50%. It is recommended as the preferred initial cholesterol-lowering strategy for secondary prevention of ASCVD in Chinese patients.

#### 5.2.3 Statins combined with a PCSK9i

Statin combination with PCSK9i can simultaneously inhibit cholesterol synthesis and enhance LDL-C clearance. This regimen can reduce LDL-C by about 75%, and by rapidly reducing LDL-C levels, LDL-C can be reached as early as possible.

The addition of a PCSK9i to a background of maximum tolerated statin therapy significantly increases both the magnitude of LDL-C reduction and the target rate (Cannon et al., 2015b). Evolocumab for Early Reduction of LDL - Cholesterol Levels in Patients with Acute Coronary Syndromes (EVOPACS) study, which enrolled hospitalized ACS patients, demonstrated that adding evolocumab early during hospitalization to a regimen of atorvastatin 40 mg/day resulted in 95.7% of patients achieving target LDL-C level after 8 weeks of treatment (Koskinas et al., 2019). In terms of reducing cardiovascular events, both the Further Cardiovascular Outcomes Research with PCSK9Inhibition in Subjects with Elevated Risk (FOURIER) and Evaluation of Cardiovascular Outcomes After an Acute Coronary Syndrome During Treatment with Alirocumab (ODYSSEY OUTCOMES) trials consistently confirmed that adding a PCSK9 inhibitor to statin therapy can reduce the risk of MACE by approximately 15% (Sabatine et al., 2015; Schwartz et al., 2018). Additionally, multiple studies have shown that addition of a PCSK9i to statin background therapy has a positive effect on coronary plaque regression (Nicholls et al., 2016; Raber et al., 2022).

When initiating statin therapy in combination with a PCSK9i, patient willingness, affordability, and cost-effectiveness should also be considered; it is only suitable for a subset of patients with very high-risk ASCVD (Wang et al., 2025). 2023 Chinese guideline for lipid management recommend that for ultra-high risk patients with a high baseline LDL-C level (LDL-C ≥4.9 mmol/L), for whom achieving the target is anticipated to be difficult with a moderate-intensity statin combined with a cholesterol absorption inhibitor, initiation of a moderate-intensity statin combined with a PCSK9i may be considered directly (Li et al., 2023). Consequently, this consensus endorses the strategy of initiating cholesterol-lowering therapy with a statin combined with a PCSK9i for ASCVD patients at ultra-high risk who present with elevated baseline LDL-C levels.

EXPERT OPINION 5

1. A combination therapy of statins and PCSK9i is recommended as an initiating cholesterol-lowering strategy for ASCVD patients at ultra-high risk who present with elevated baseline LDL-C levels (LDL-C ≥4.9 mmol/L).

#### 5.2.4 Cholesterol absorption inhibitors combined with PCSK9i

Combination therapy with a cholesterol absorption inhibitor and a PCSK9i simultaneously reduces cholesterol absorption and increases LDL-C clearance, achieving an approximate 70% reduction in LDL-C (Mach et al., 2025). Although the combination of a cholesterol absorption inhibitor and a PCSK9i is not a commonly used clinical treatment regimen for initiating cholesterol-lowering therapy, it is not uncommon to consider this approach in cases of statin intolerance. Especially for ASCVD patients who are statin intolerant, initiating the combination of a cholesterol absorption inhibitor and a PCSK9i can help achieve LDL-C targets as soon as possible. In the Goal Achievement after Utilizing an anti-PCSK9 antibody in Statin Intolerant Subjects (GAUSS) and GAUSS-2 studies showed that in patients with statin intolerance, evolocumab combined with ezetimibe reduced LDL-C by up to 63% from baseline, and the overall incidence of adverse reactions was similar to that of monotherapy (Stroes et al., 2014; Sullivan et al., 2012).

Therefore, for statin-intolerant ASCVD patients, the combination of cholesterol absorption inhibitor and PCSK9i represents an effective and well-tolerated option for initial cholesterol-lowering therapy.

#### 5.2.5 Moderate-intensity statins combined with cholesterol absorption inhibitors and PCSK9i

As mentioned above, statins, cholesterol absorption inhibitors and PCSK9i respectively act on the synthesis, absorption and clearance of cholesterol, forming a comprehensive cholesterol-lowering therapeutic cycle. A network meta-analysis demonstrated that the addition of a PCSK9i to moderate-intensity statin combined with ezetimibe can further reduce LDL-C by approximately 50%–60% compared to placebo (Liu et al., 2024).

Studies have confirmed that initiating cholesterol-lowering therapy with a combination of moderate-intensity statin, cholesterol absorption inhibitor, and PCSK9i can achieve a total LDL-C reduction of 70%–80% or more (Liu and Han, 2022; Masana et al., 2020). A retrospective study in China showed that triple therapy with rosuvastatin 10 mg/day, ezetimibe 10 mg/day, and evolocumab 140 mg every 2 weeks reduced LDL-C by 73.59% in patients with coronary heart disease (Liu and Han, 2022).

Therefore, for ultra high-risk ASCVD patients elevated baseline LDL-C levels (LDL-C ≥4.9 mmol/L) and those with FH, this consensus recommends the use of moderate-intensity statin combined with a cholesterol absorption inhibitor (such as ezetimibe) and a PCSK9i as a preferred initial cholesterol-lowering strategy.

#### 5.2.6 The maximum tolerated statins combined with cholesterol absorption inhibitors and PCSK9i

High-intensity or maximum tolerated-dose statins combined with a cholesterol absorption inhibitor and PCSK9i can also be considered as an initial cholesterol-lowering treatment strategy in some patients. One RCT showed that in post-PCI patients, triple therapy with evolocumab, high-intensity atorvastatin, and ezetimibe reduced LDL-C levels from 3.5 mmol/L to 0.6 mmol/L after 1 month of treatment. Compared to the control group (high-intensity atorvastatin combined with ezetimibe), this regimen significantly reduced LDL-C levels (−83.9% vs. −63.9%) and the incidence of MACE, with no significant difference in the incidence of adverse reactions (Hao et al., 2022). A network meta-analysis including 14 RCTs showed that in high-risk or very high-risk ASCVD patients, maximum tolerated-dose statin combined with ezetimibe and PCSK9i reduced the risk of non-fatal MI and non-fatal stroke, but in moderate- and low-risk patients, the triple therapy provided only slight or no additional benefit in reducing non-fatal MI or non-fatal stroke (Khan et al., 2022). Therefore, for ultra high-risk ASCVD patients with elevated baseline LDL-C, early addition of a PCSK9i to the treatment regimen may rapidly lower LDL-C levels and improve cardiovascular outcomes without increasing the risk of adverse events.

Based on the fact that high-intensity or maximum tolerated dose statins are mainly recommended strategies in European and American guidelines, this consensus recommends that patients with FH may consider maximum tolerated dose statin combined with cholesterol absorption inhibitor and PCSK9i as the initial cholesterol-lowering therapy.

EXPERT OPINION 6

1. A combination therapy of moderate-intensity statins, cholesterol-absorption inhibitors (e.g., ezetimibe) and PCSK9i is an appropriate initial regimen for ultra-high-risk ASCVD patients who present with markedly elevated baseline LDL-C and for those with FH. This triple strategy reliably achieves lipid targets and is well tolerated.

#### 5.2.7 Statins combined with cholesterol absorption inhibitors, PCSK9i, and lipoprotein apheresis

Lipoprotein Apheresis (LA) is an extracorporeal therapy that selectively removes LDL-C and lipoprotein(a) [Lp(a)] from the circulation. This procedure achieves an average LDL-C reduction of 55% and an Lp(a) reduction exceeding 50%. It can also improve clinical manifestations, such as xanthomas, in patients with familial hypercholesterolemia (FH) and is generally well-tolerated (Cuchel et al., 2023; Gianos et al., 2024; Pottle et al., 2019). Its primary application is for FH patients, particularly those with homozygous FH (HoFH) and elevated Lp(a) (Gianos et al., 2024). For HoFH patients, combination therapy of lipid-lowering drugs and LA is the cornerstone of cholesterol-lowering treatment. A study showed that, under the background of maximum tolerated cholesterol-lowering therapy (including statins and/or ezetimibe), the addition of LA reduced LDL-C by 69% in HoFH patients and by 72% in heterozygous FH (HeFH) patients. Subsequently, 87.5% of patients who additionally use PCSK9i therapy further reduced LDL-C levels and reduced the frequency of LA (Page et al., 2021). Therefore, HoFH patients can consider initiating a triple-combination therapy with a moderate- or high-intensity statin, a cholesterol absorption inhibitor, and a PCSK9i. If LDL-C still does not achieve the target, LA can be added (Tromp et al., 2022). LA combined with the triple-combination therapy, may also be used in the initial treatment of refractory HeFH. However, the use of this quadruple-combination strategy as an initial cholesterol-lowering treatment strategy currently lacks supporting evidence from randomized controlled trials.

#### 5.2.8 Statins combined with cholesterol absorption inhibitors, PCSK9i, and probucol

Probucol works by incorporating into LDL particles, thereby enhancing LDL metabolism, and has a significant lipid-lowering effect in patients with HeFH and HoFH. In patients with asymptomatic hypercholesterolemia, probucol monotherapy can reduce LDL-C by 29% (Sawayama et al., 2002).

A Japanese cohort study showed that, in HeFH secondary prevention patients (94.6% of whom were already on lipid-lowering therapy such as statins), the use of probucol over a median follow-up of 10 years significantly reduced the risk of first cardiovascular events by 87%. However, no significant reduction in cardiovascular event risk was observed with long-term probucol treatment in primary prevention patients (Yamashita et al., 2008). Furthermore, the use of probucol significantly reduced the xanthoma scores and xanthoma regressed in 36% of patients (Fujita and Shirai, 1996). Therefore, based on the clinical severity of FH and the extent of LDL-C elevation, initiation of a quadruple-combination therapy involving a statin, a cholesterol absorption inhibitor, a PCSK9i, and probucol may be considered for FH patients, particularly for those with HoFH accompanied by xanthomas. In addition, it is necessary to pay attention to the effect of probucol in reducing HDL-C and risk of QT prolongation.

### 5.3 Novel cholesterol-lowering therapy regimen

#### 5.3.1 Bempedoic acid

Bempedoic acid is a novel non-statin lipid-lowering drug that inhibits hepatic cholesterol synthesis by targeting ATP-citrate lyase (ACL), an enzyme upstream of HMG-CoA reductase. Bempedoic acid monotherapy can reduce LDL-C by 21%–30% and has been shown to lower risk of major cardiovascular events (Laufs et al., 2019; Nissen et al., 2023; Thompson et al., 2016). Since it is not metabolized into active products in skeletal muscle, bempedoic acid may avoid the skeletal muscle-related adverse effects associated with statin therapy (Pinkosky et al., 2016). But caution is still warranted about the adverse reactions such as hyperuricemia and elevated liver enzymes (Goldberg et al., 2019). Approved by FDA in 2020 (Markham, 2020). This regimen is recommended as an initial cholesterol-lowering strategy primarily for statin-intolerant patients with hypercholesterolemia (including HeFH) (Ray et al., 2019).

#### 5.3.2 Bempedoic acid combined with cholesterol absorption inhibitors

In a Phase II clinical trial, bempedoic acid combined with ezetimibe reduced LDL-C by 48% (Thompson et al., 2016). The CLEAR Tranquility study demonstrated that adding bempedoic acid to ezetimibe further reduced LDL-C by 28.5% in statin-intolerant patients (Ballantyne et al., 2018). This combination therapy may be considered as an initial cholesterol-lowering strategy for ASCVD high-risk patients with statin intolerance.

#### 5.3.3 Bempedoic acid combined with PCSK9i

A Phase II study showed that the addition of bempedoic acid to PCSK9i therapy can further reduce LDL-C by 30%, and significantly lower apolipoprotein B (ApoB), non-high-density lipoprotein cholesterol (non-HDL-C), total cholesterol (TC), and high-sensitivity C-reactive protein (hsCRP) levels, with good tolerability (Rubino et al., 2021). This combination therapy may be considered as an initial cholesterol-lowering strategy for statin-intolerant, ultra high-risk ASCVD patients with elevated baseline cholesterol levels.

#### 5.3.4 Maximum tolerated statins combined with bempedoic acid and cholesterol absorption inhibitors

A study has shown that in high-risk patients with ASCVD, HeFH, or multiple CVD risk factors, the combination of bempedoic acid and ezetimibe on the basis of the maximum tolerated dose of statins can further reduce LDL-C by 36%, with good tolerability and a patient adherence rate exceeding 80% (Ballantyne et al., 2020). This combination therapy may be considered as an initial cholesterol-lowering strategy for high-risk patients with ASCVD, HeFH, or multiple CVD risk factors.

#### 5.3.5 Evinacumab

Evinacumab is a monoclonal antibody targeting angiopoietin-like protein 3 (ANGPTL3). It reduces the conversion of very low-density lipoprotein (VLDL) to LDL-C by inhibiting ANGPTL3, thereby lowering LDL-C levels (Ruscica et al., 2020). A Phase III trial showed that evinacumab treatment in HoFH patients significantly reduced LDL-C by 47.1% compared to placebo for 24 weeks, with a comparable incidence of adverse events between groups (Raal et al., 2020). A real-world study showed that long-term evinacumab adjunctive to lipid-lowering therapy can improve cardiovascular event-free survival of patients with HoFH, however, randomized controlled trial assessing the effect of evinacumab on cardiovascular outcomes is not yet available (Béliard et al., 2024). Approved by the FDA for the treatment of HoFH in adults and pediatric patients, with the pediatric indication expanded from children aged 5 years and older in 2023 to those aged 1 year and older in 2025, evinacumab may be considered as an initial cholesterol-lowering strategy for this patient population. The latest ESC guidelines have also emphasized the use of this drug in patients with HoFH.

#### 5.3.6 Lomitapide

Lomitapide is a microsomal triglyceride transfer protein (MTP) inhibitor that reduces LDL-C levels by inhibiting MTP activity, thereby decreasing the synthesis and secretion of VLDL and chylomicrons in the liver and intestine. Lomitapide treatment can reduce LDL-C by approximately 53% in HoFH patients, with adverse events mostly being mild gastrointestinal reactions and liver-related adverse effects (Blom et al., 2024; Masana et al., 2024). Approved in the United States for the treatment of HoFH for children aged 2 years and older, lomitapide may be considered as an initial cholesterol-lowering therapeutic option for HoFH patients.

The selection of initial cholesterol-lowering strategies for different populations is detailed in Table 3.

EXPERT OPINION 7

1. The initial treatment strategies options for HoFH patients include statins combined with cholesterol absorption inhibitors and PCSK9i; statins combined with cholesterol absorption inhibitors, PCSK9i, and LA; or statins combined with cholesterol absorption inhibitors, PCSK9i, and probucol. And if accessible, evinacumab and lomitapide may also be considered in initial combination therapy.
2. For statin-intolerant patients, based on baseline LDL-C levels and ASCVD risk stratification, initial alternative therapy may include: cholesterol absorption inhibitors; cholesterol absorption inhibitors combined with PCSK9i; bempedoic acid; bempedoic acid combined with cholesterol absorption inhibitors, or bempedoic acid combined with PCSK9i.

**TABLE 3:** Overview of initial cholesterol-lowering treatment strategies.

Regimen	LDL-C reduction	Target population	CV Risk reduction evidence^a^	Considerations
Monotherapy				
High-intensity/maximum tolerated statins	≥50%	High/very high-risk ASCVD patients (Western populations)	Yes	Myopathy, liver enzyme elevation, new-onset diabetes; higher statin intolerance risk in Asians
Moderate-intensity statins	25%–50%	Primary prevention of ASCVD	Yes	Limited LDL-C reduction
Cholesterol absorption inhibitors	18%–20%	Statin-intolerant patients or elderly (≥75 years) in primary prevention	Yes	Limited LDL-C reduction
PCSK9i	45%–57%	Primary prevention in statin-intolerant patients or those concerned about statin side effects	No	High cost; lacks CV outcome evidence as monotherapy; adherence concerns with long-term use
Combination therapy				
High-intensity/maximum tolerated statins combined with cholesterol absorption inhibitors	60%–65%	High/very high-risk ASCVD patients (Western populations); FH patients failing to meet the target on moderate-intensity statin combined with cholesterol absorption inhibitor	Yes	Higher statin intolerance risk in Asians
Moderate-intensity statins combined with cholesterol absorption inhibitors	50%–60%	ASCVD patients; primary prevention with baseline LDL-C ≥4.9 mmol/L or FH; high-risk diabetes or severe CKD	Yes	Lower goal attainment in ultra-high risk ASCVD
Statins combined with PCSK9i	∼75%	Ultra-high risk ASCVD with high baseline LDL-C (≥4.9 mmol/L)	Yes	Long-term adherence and statin-related side effects
Cholesterol absorption inhibitors combined with PCSK9i	∼70%	Statin-intolerant very high risk and ultra-high risk ASCVD patients	No	Limited evidence
Statins combined with cholesterol absorption inhibitors and PCSK9i	70%–80%	Ultra-high risk ASCVD or FH with high baseline LDL-C (≥4.9 mmol/L)	Yes	Polypharmacy; adherence concerns
Statins combined with cholesterol absorption inhibitors and PCSK9i and lipoprotein apheresis	Lack of data	HoFH, refractory HeFH	No	Polypharmacy; adherence concerns; limited evidence
Statins combined with cholesterol absorption inhibitors and PCSK9i and probucol	Lack of data	FH patients, particularly HoFH with xanthomas	No	Polypharmacy; adherence concerns; limited evidence; HDL-C-lowering effects; QT prolongation
Novel agents (not yet approved in China)				
Bempedoic acid	21%–30%	Statin-intolerant hypercholesterolemia (including HeFH)	Yes	Hyperuricemia; hepatotoxicity
Bempedoic acid combined with cholesterol absorption inhibitors	48%	Statin-intolerant high-risk ASCVD patients	No	Hepatotoxicity, gastrointestinal side effects
Bempedoic acid combined with PCSK9i	70%–80%	Statin-intolerant and ultra-high risk ASCVD with high baseline cholesterol	No	Limited evidence
Maximum tolerated statins combined with bempedoic acid and cholesterol absorption inhibitors	68%	High-risk patients with ASCVD, HeFH, and those with multiple CVD risk factors	No	Polypharmacy; adherence concerns; adverse effect monitoring required
Evinacumab	47%	HoFH	No	Lacks large real-world studies
Lomitapide	53%	HoFH	No	Lacks large real-world studies

ASCVD, atherosclerotic cardiovascular disease; LDL-C, low-density lipoprotein cholesterol; PCSK9i, Proprotein Convertase Subtilisin/Kexin Type 9 Inhibitor; FH, familial hypercholesterolemia; HoFH, homozygous familial hypercholesterolemia; HeFH, heterozygous familial hypercholesterolemia; cholesterol; CKD, chronic kidney disease.

^a^ Only evidence regarding this regimen as initial cholesterol-lowering therapy was retrieved. This table is for clinical use only.

## 6 Clinical implementation recommendations for initial cholesterol-lowering strategies

### 6.1 Monitoring during treatment

According to ASCVD risk stratification, physicians should monitor whether blood lipid parameters reach target levels and assess adverse drug reactions and medication adherence in patients. At the initiation of therapy, blood lipids (such as LDL-C, non-HDL-C, triglyceride, and HDL-C), blood glucose (such as fasting glucose or Glycated hemoglobin), liver enzymes (Alanine aminotransferase, Aspartate Transferase), and creatine kinase (CK) should be re-evaluated 4–6 weeks after starting medication. If after cholesterol-lowering treatment, all lipid parameters reach target levels and no adverse drug reactions are observed, the monitoring interval may be gradually extended to every 3–6 months. For patients at very high risk or ultra-high risk, more frequent monitoring is required. Additionally, patient adherence should be assessed by inquiring about medication regularity, self-initiated dose reductions or discontinuations, and the impact of potential side effects (such as myalgia or gastrointestinal reactions) on treatment persistence.

### 6.2 Strategies to improve adherence

1) Selection of Appropriate Medications and Initial Regimens: Simplify treatment plans by reducing pill burden and dosing frequency, such as utilizing FDCs. A German retrospective study showed that compared to free-equivalent combination (FEC), FDC of statin and ezetimibe resulted in a greater reduction in LDL-C (19.4% vs. 28.4%) and significantly increased the target attainment rate by 10.5% (Katzmann et al., 2022). Data from the Italian healthcare database further indicated that FDC improved medication adherence and reduced the risk of treatment discontinuation compared to FEC (Rea et al., 2021). 2) Development of Individualized Treatment Plans: Formulate cholesterol-lowering treatment strategy based on the patient’s specific clinical profile (e.g., comorbidities, drug tolerability) to improve adherence. 3) Strengthen Patient Management: Implement patient education and primary care management strategies, explain in detail to patients the risks of hypercholesterolemia, the necessity of cholesterol-lowering therapy, and the importance of long-term adherence. For example, by providing written materials or popular science videos on cholesterol treatment, *etc.*, to improve patient understanding of the disease and its treatment. 4) Strengthening Physician-Patient Communication: Establish a strong physician-patient relationship, conduct regular follow-ups to address patient concerns and worries, and provide thorough explanations to increase patient trust.

## 7 Summary and future perspectives

Choosing an appropriate initial cholesterol-lowering strategy is critical for achieving early and long-term lipid control and reducing treatment delays. The high-intensity or maximally tolerated statin strategy has been a primary early treatment option. However, in recent years, with the continuous advent of non-statin drugs, especially the robust clinical trial results for cholesterol absorption inhibitors (such as ezetimibe) and PCSK9i, the era of “post-statin” cholesterol-lowering therapy has begun. Current cholesterol-lowering strategies have gradually shifted from a stepwise approach to initial combination therapy to achieve dual lipid targets (both absolute LDL-C values and percentage reduction) as soon as possible. For very high risk or ultra-high risk ASCVD patients, especially Chinese patients, initiating combination therapy with moderate-intensity statin and ezetimibe, rather than high-intensity statin monotherapy, can bring more significant cardiovascular benefits under the premise of better lipid goal attainment rates, adherence and safety. Moreover, oral administration is more convenient, with better adherence and cost-effectiveness, making it an ideal initial cholesterol-lowering strategy for Chinese ASCVD patients.

The other initial cholesterol-lowering treatment strategies summarized in this consensus are helpful for the implementation of individualized strategies for cholesterol-lowering therapy in clinical practice, and play a positive guiding role in improving the target attainment rate of cholesterol-lowering treatment in the real world.

## Group members of cardiovascular metabolic medicine task group, national cardiovascular disease expert committee

Hong Chen, Peking University People’s Hospital; Zhen-Yue Chen, Ruijin Hospital, Shanghai Jiao Tong University School of Medicine; Xiang Cheng, Union Hospital affiliated to Tongji Medical College of Huazhong University of Science and Technology; Ke-Fei Dou, Ying Gao, Yuan-Lin Guo, Jian-Jun Li, Xiao Wang, Na-Qiong Wu, Jin-Gang Yang, Cheng-Gang Zhu, Fuwai Hospital, Chinese Academy of Medical Sciences; Kai Huang, Renmin Hospital of Wuhan University; Li-Wen Li, Guangdong People’s Hospital; Yong Li, Huashan Hospital, Fudan University; Chun Liang, Shanghai Changzheng Hospital; Dao-Quan Peng and Zhi-Guang Zhou, Second Xiangya Hospital of Central South University; Ping Ye, Chinese PLA General Hospital; Da-Qing Zhang, China Medical University; Dong Zhao, Beijing Anzhen Hospital, Capital Medical University; Jia-Jun Zhao, Shandong First Medical University.
