# Supplementary material for: Chinese expert consensus on the choice of initial cholesterol-lowering strategy
Source: Front Pharmacol. 2026 May 19;17:1798787. doi: 10.3389/fphar.2026.1798787 (PMC13226597; doi:10.3389/fphar.2026.1798787)
Supplement: Supplementary file 1 [file Supplementaryfile1.docx]

**Table S1. Clinical evidence for maximal tolerated/high-intensity statin therapy**

| **Study name** | **Study design** | **Participants** | **Treatment** | **Primary outcome** | **Followup**  **duration** | **Key efficacy/effectiveness**  **results** | **Adverse reactions** | **Treatment adherence** |
| --- | --- | --- | --- | --- | --- | --- | --- | --- |
| **MIRACL**(Schwartz et al., 2001) | Randomized, double-blind | ACS patients (N=3,086) | Atorvastatin 80 mg/day or placebo initiated within 24-96 h | Death, non-fatal MI, resuscitated cardiac arrest, or recurrent symptomatic myocardial ischemia | 16 weeks | Primary endpoint: Atorvastatin vs Placebo (14.8% vs 17.4%; RR 0.84, 95% CI 0.70-1.00, P=0.048).  **LDL-C at 6 weeks:** 1.9 mmol/L vs 3.5 mmol/L. | Elevated liver enzymes (>3× ULN): 2.5% vs 0.6% | Not reported |
| **PROVE IT-TIMI 22**(Rouleau, 2005) | RCT | ACS patients (N=4,162) | Intensive (Atorvastatin 80 mg/day) vs Standard (Pravastatin 40 mg/day) | The first occurrence of any of the following events: death,  MI, documented  unstable angina  requiring hospital  admission, coronary  artery revascularizationb (occurring  at least 30 days  after randomization) or stroke | Median 2 years | Primary endpoint: Intensive vs Standard (22.4% vs 26.3%; 16% RRR, 95% CI 5%-26%, P=0.005) | CK >3× ULN: 1.5% vs 1.1% | Discontinuation (any reason): 30.4% vs 33.0%. Discontinuation (myalgia/CK): 3.3% vs 2.7% |
| **TNT**(LaRosa et al., 2005) | RCT | Stable CHD patients (N=10,001) | Atorvastatin 80 mg/day vs Atorvastatin 10 mg/day | Time to first major CV event: CHD death, non-fatal non-procedural MI, resuscitated cardiac arrest, or fatal/non-fatal stroke | Median 4.9 years | Major CV events: Atorvastatin 80 mg vs Atorvastatin 10 mg (8.7% vs 10.9%; 22% RRR, HR=0.78, 95% CI 0.69-0.89, P<0.001) | Liver enzymes (>3× ULN): 1.2% vs 0.2% (P<0.001); Muscle-related pain: 14.8% vs 4.7% | Discontinuation (AE-related): 7.2% vs 5.3% (P<0.001) |
| **SPARCL**(Amarenco et al., 2006) | Randomized, double-blind | Stroke or TIA patients (N=4,731) | Atorvastatin 80 mg/day vs Placebo | The time when the first non-fatal or fatal stroke occurred | Median 4.9 years | Primary endpoint: Atorvastatin vs Placebo (11.2% vs 13.1%; 16% RRR, HR=0.84, 95% CI 0.71-0.99, P=0.03) | Liver enzymes (>3× ULN): 2.2% vs 0.5% (P<0.001) | Not reported |

MIRACL: Myocardial Ischemia Reduction with Aggressive Cholesterol-lowering; PROVE IT-TIMI 22: Pravastatin or Atorvastatin Evaluation and Infection Therapy–Thrombolysis in Myocardial Infarction 22; TNT: Treating to New Targets ; SPARCL: Stroke Prevention by Aggressive Reduction in Cholesterol Levels; ACS: Acute Coronary Syndrome; MI: Myocardial Infarction; LDL-C: Low-Density Lipoprotein Cholesterol; TIA: Transient Ischemic Attack; CK: Creatine Kinase; RCT: Randomized Controlled Trial; CHD: Coronary Heart Disease; CV, Cardiovascular; RRR, Relative Risk Reduction; HR, Hazard Ratio; CI, Confidence Interval; ULN, Upper Limit of Normal; AE, Adverse Event..

**Table S2. Clinical evidence for moderate-intensity statin therapy**

| **Study name** | **Study design** | **Participants** | **Treatment** | **Primary outcome** | **Followup**  **duration** | **Key efficacy/effectiveness**  **results** | **Adverse reactions** | **Treatment adherence** |
| --- | --- | --- | --- | --- | --- | --- | --- | --- |
| **WOSCOPS**(Shepherd et al., 1995) | Randomized, double-blind | Men with hypercholesterolemia and no prior history of coronary heart disease (N=6,595) | Pravastatin 40 mg/day or Placebo | Primary endpoint, Non-fatal MI or coronary heart disease death | Median 4.9 years | Primary endpoint: Pravastatin vs Placebo (5.5% vs 7.9%; 31% risk reduction, 95% CI 17%-43%; P<0.001) | Myalgia: 20 vs 19 cases;  Muscle soreness: 97 vs 102 cases;  Elevated liver transaminases (>3 × ULN): 20 vs 12 cases | Not reported |
| **Large-Scale RCT**(Collins et al., 2004) | RCT | Patients with cerebrovascular disease or other high-risk conditions (N=20,536) | Simvastatin 40 mg/day or matching Placebo | First major vascular event, non-fatal MI, coronary heart disease death, any stroke, or any revascularization | 5 years | New stroke: Simvastatin vs Placebo (4.3% vs 5.7%; 25% risk reduction, 95% CI 15%-34%; P<0.0001);  Major vascular events: 24.7% vs 29.8%; 20% reduction, 95% CI 8%-29%; P=0.001 | Cognitive function: 23.7% vs 24.2% | Not reported |
| **CHILLAS**(Zhao et al., 2014) | Randomised, open-label trial | ACS patients (N=1,355) | Moderate-dose statin (Atorvastatin 10 mg/day or equivalent) vs Double-dose statin (Atorvastatin 20-40 mg/day or equivalent) | Primary endpoint, Cardiac death, non-fatal MI, revascularization, ischemic stroke, documented unstable angina, or severe heart failure requiring urgent hospitalization | 2 years | Major CV events: Moderate-dose statin vs Double-dose statin (3.9% vs 5.5%; HR=1.39, 95% CI 0.78-2.46, P=0.261) | Elevated serum aminotransferases (>3× ULN): 10 vs 13 cases; P < 0.001  Elevated serum creatine kinase (>5× ULN): 1 vs 2 cases | Not reported |
| **CCSPS**(Lu et al., 2008) | Randomized, double-blind | Patients with prior myocardial infarction (N=4,810) | Xuezhikang 600 mg twice daily or Placebo | Primary endpoint, major coronary events, including non-fatal MI and coronary heart disease death | Average 4.5 years | Primary endpoint: Xuezhikang vs Placebo (5.7% vs 10.4%; 45% relative risk reduction, P<0.001) | No treatment-related serious adverse events or deaths reported | Treatment discontinuation due to adverse events was similar between groups |

WOSCOPS: West of Scotland Coronary Prevention Study; CHILLAS: China Intensive Lipid Lowering with Statins in Acute Coronary Syndrome; CCSPS China Coronary Heart Disease Secondary Prevention Study.

**Table S3. Clinical evidence for moderate-intensity statin combined with cholesterol absorption inhibitor**

| **Study name** | **Study design** | **Participants** | **Treatment** | **Primary outcome** | **Followup**  **duration** | **Key efficacy/effectiveness**  **results** | **Adverse reactions** | **Treatment adherence** |
| --- | --- | --- | --- | --- | --- | --- | --- | --- |
| **SHARP**(Baigent et al., 2011) | Randomized, double-blind | Patients with CKD patients and no known history of MI or coronary revascularization (N=9,270) | Group 1: Simvastatin 20 mg/day combined with ezetimibe 10 mg/day  Group 2: Simvastatin 20 mg/day → Simvastatin 20 mg/day combined with ezetimibe 10 mg/day  Group 3: Placebo | First major atherosclerotic event (non-fatal MI, coronary death, non-hemorrhagic stroke, or arterial revascularization) | Median 4.9 years | First major atherosclerotic event: Combination vs Placebo: 11.3% vs 13.4% (RR 0.83, 95% CI 0.74-0.94, P=0.0021) | Muscle pain: 21.3% vs 20.8%  > 10 to ≤40 × ULN, 0.4% vs 0.3%  > 40 × ULN, 0.1% vs 0.1%  Myopathy: 0.2% vs 0.1%  Rhabdomyolysis: 0.1% vs 0  Persistently increased transaminases: 0.6% vs 0.6% | Treatment discontinuation: 33.0% vs 36.1%  Compliance (receiving ≥ 80% scheduled treatment): 77% vs 3% at the first year, 68% vs 14% at the fourth year |
| **IMPROVE-IT**(Cannon et al., 2015) | Randomized, double-blind | Patients hospitalized for ACS (N=18,144) | Group 1: Simvastatin 40 mg/day combined with Ezetimibe 10 mg/day  Group 2: Simvastatin 40 mg/day combined with placebo | A composite of death from cardiovascular disease, a major coronary events (non-fatal MI, documented unstable angina requiring hospital admission, or coronary revascularization), or non-fatal stroke | Median 6 years | Kaplan-Meier event rate for the primary endpoint at 7 years: simvastatin combined with ezetimibe vs. simvastatin combined with placebo (32.7% vs. 34.7%; HR 0.936, 95% CI 0.89-0.99, P=0.016) | Myopathy: 0.2% vs 0.1%  Rhabdomyolysis: 0.1% vs 0.2%  Rhabdomyolysis, myopathy, myalgia with creatine kinase elevation ≥ 5 × ULN, 0.6% vs 0.6%  ALT, AST or both ≥ 3 × ULN, 2.5% vs 2.3% | Treatment discontinuation due to AEs: 10.6% vs 10.1% |
| **PL-ACS**(Lewek et al., 2023) | National, Prospective, Observational registry study | Patients hospitalized with ACS (N=1,536) | Propensity score-matched group: Initial combination therapy of statin and ezetimibe vs. statin monotherapy | All-cause mortality | 3 years | Kaplan-Meier event rates for all-cause mortality: Combination 3.5% vs monotherapy 5.9% (P=0.041)  2 years: 4.3% vs 7.8% (P=0.019）  3 years: 5.5% vs 10.2% (OR 0.526，95%CI 0.378-0.733，P=0.024) | Not reported | Not reported |
| **RACING**(Kim et al., 2022) | Randomised, open-label, non-inferiority trial | ASCVD patients (N=3,780) | Group 1: Rosuvastatin 10 mg/day combined with Ezetimibe 10 mg/day  Group 2: Rosuvastatin 20 mg/day | The occurrence of cardiovascular death, major cardiovascular events, or non-fatal stroke within 3 years | Median 3 years | Primary endpoint: Combination 9.1% vs monotherapy 9.9% (absolute difference: 0.78%，95%CI -2.39-0.83)  LDL-C<70mg/dL:  1 years: 73% vs 55%,  2 years: 75% vs 60%,  3 years: 72% vs 58% (all P<0.0001） | Muscle-related AEs: 1.1% vs 1.9%  Myalgia: 0.9% vs 1.6%  Myopathy: 0.1% vs 0.2%  New-onset diabetes: 7.9% vs 8.7%  New-onset diabetes with anti-diabetic medication initiation: | Treatment discontinuation or dose reduction due to AEs or intolerance: 4.8% vs 8.2% (P<0.0001) |
| **ROSETTA-Stroke**(Hong et al., 2023) | Randomized, Open-label, Controlled trial | Patients with recent ischemic stroke < 90 days (N=584) | Group 1: Rosuvastatin 10 mg/day combined with Ezetimibe 10 mg/day  Group 2: Rosuvastatin 20 mg/day | LDL-C reduction ≥50% from baseline at 90 days | 90 days | LDL-C reduction ≥ 50% from baseline at 90 days: Combination 72.5% vs monotherapy 57.6% (OR 1.944，95%CI 1.352-2.795，P=0.0003)  Multiple lipid goal achievement: 71.1% vs 53.7% (P<0.0001)  Major vascular events: 0.4% vs 3.5% (OR 0.101，95%CI 0.013-0.805，P=0.0091) | Myalgia: 2.3% vs1.4%  No clinically Significant myopathy | Treatment interruption or discontinuation: 1.5% vs 3.9% |
| **CONNECT DES**(Lee et al., 2023) | Observational study based on a nationwide cohort database | Patients who were prescribed rosuvastatin after drugeluting stent implantation (N=72,050) | Groups assigned after stabilized inverse probability of treatment weighting: Rosuvastatin 10 mg/day combined with ezetimibe 10 mg/day vs. Rosuvastatin 20 mg/day | 3-year composite event of cardiovascular death, MI, coronary artery revascularization, hospitalization for heart failure treatment or non-fatal stroke | 3 years | Primary endpoint: Combination 11.6% vs monotherapy 15.2% (HR 0.75, 95%CI 0.70-0.79，P<0.001) | New-onset diabetes requiring medication: 7.7% vs 9.6% (HR 0.80, 95%CI 0.72-0.88, P<0.001） | Statin discontinuation: 6.5% vs 7.6% (HR 0.85, 95%CI 0.78-0.94, P<0.001) |
| **Nationwide cohort study**(Jun et al., 2024) | Propensity-matched nationwide cohort study based on the Korean National Health Insurance Service datasets | Individuals with dyslipidaemia without preexisting CVDs (N=92,948) | Propensity score-matched group: Low-/moderate-intensity statin combined with ezetimibe vs. high-intensity statin monotherapy | A composite of incident MI, stroke, and all-cause death | Median 3.3 years | Primary endpoint: Combination 7.3/1000 person-years vs monotherapy 11.1/1000 person-years (HR 0.84, 95%CI 0.77-0.92，P<0.001) | Not reported | Difference in statin adherence not significant |
| **Nationwide cohort study**(Park et al., 2024) | Propensity-matched nationwide cohort study based on the Korean National Health Insurance Service datasets | Patients with T2DM and dyslipidemia (N=27,221) | Propensity score-matched group: Low-/moderate-intensity statin combined with ezetimibe vs. high-intensity statin monotherapy | Incidence of Composite outcomes of MI, stroke, and all-cause death | 4 years | Incidence of composite outcomes: HR 0.85, 95%CI 0.74-0.98  LDL-C: 74±37.9 vs 80.8±38.8 mg/dL (P<0.001） | Not reported | Not reported |
| **Nationwide cohort study**(Jang et al., 2024) | Propensity-matched nationwide cohort study based on the Korean National Health Insurance Service datasets | Patients who underwent percutaneous coronary intervention with ACS (N=21,446) | Propensity score-matched group: Moderate-intensity statin combined with ezetimibe vs. high-intensity statin monotherapy | A composite of MI, ischaemic stroke and all-cause mortality | Mean 1028 vs 1026 days | Primary endpoint: Combination 4.29 events per 100 person-years vs monotherapy 4.73 events per 100 person-years (HR 0.85, 95%CI 0.78-0.92) | Not reported | Treatment adherence (≥ 80% for 3 months): 67.8% vs 50.0% (P<0.001) |
| **MESIA**(Lv et al., 2024) | RCT | Acute ischemic cerebrovascular disease (N=382) | Group 1: Atorvastatin 10-20 mg/day or Rosuvastatin 5-10 mg/day combined with Ezetimibe 10 mg/day  Group 2: Atorvastatin 40 mg/day or Rosuvastatin 20 mg/day | LDL-C levels and achievement rate at 3 months | 3 months | Primary endpoint: LDL-C achievement rate, Combination 89.9% vs. monotherapy 70.8%, P=0.005  LDL-C: -55.6% vs. -48.0%, P=0.001 | Musculoskeletal adverse events: 1.5% vs. 3.1%  No treatment discontinuations due to adverse events or serious adverse events occurred | Not reported |
| **Nationwide cohort study**(Choo et al., 2024) | Propensity-matched nationwide cohort study based on the Korean National Health Insurance Service datasets | Patients with PCI (N=21,446) | Group 1: Moderate-intensity statin combined with ezetimibe  Group 2: High-intensity statin | A composite of all-cause mortality, Coronary Revascularization, or ischaemic stroke | 2.7 years | Primary endpoint: Combination 31.9% vs. monotherapy 33.8% (HR：0.96，95%CI 0.89-1.03, P = 0.27) | New-onset diabetes: 10.7% vs. 12.5%, P=0.02 | Not reported |
| **RCT**(Cha et al., 2025) | Prospective, Multicenter, Open-label trial | ASCVD patients ≥70 years (N=561) | Group 1: Rosuvastatin 5 mg/day combined with Ezetimibe 10 mg/day  Group 2: Rosuvastatin 20 mg/day | Incidence of statin-associated muscle symptoms and LDL-C levels | 6 months | Primary endpoint: muscle symptom rate, Combination 0.7% vs. monotherapy 5.7%, P=0.005 | Significantly lower muscle symptom rate in combination group | Not reported |
| **Nationwide cohort study**(Lee et al., 2025) | Propensity-matched nationwide cohort study based on the Korean National Health Insurance Service datasets | ACS with PCI on atorvastatin (N=31,993) | Propensity score-matched group: Atorvastatin 20 mg/day combined with Ezetimibe 10 mg/day vs. Atorvastatin 40-80 mg/day | Composite endpoint of cardiovascular death, MI, coronary revascularization, hospitalization for heart failure, or non-fatal stroke | Median 3 years | Primary endpoint: Combination 12.9% vs monotherapy 15.1% (HR 0.81, 95%CI 0.74-0.88, P＜0.001) | Rhabdomyolysis: 0.2% vs 0.3%  New-onset diabetes requiring medication: 7.0% vs 8.8% | Treatment discontinuation due to AEs: 8.4% vs 10.0% (P<0.001) |
| **Nationwide cohort study** (Sohn et al., 2025) | Propensity-matched nationwide cohort study based on the Korean National Health Insurance Service datasets | Treatment-naive diabetic adults (N=31,552) | Primary prevention cohort without cardiovascular disease (high-intensity statin vs. low-intensity statin combined with ezetimibe, n=21,458)  Secondary prevention cohort with cardiovascular disease (high-intensity statin vs. low-intensity statin combined with ezetimibe, n=10,094) | Composite endpoint of MI, stroke, or cardiovascular death | Average 5.5 years | Primary endpoint:  Primary prevention cohort: intensity statin 4.85/1,000 person-years vs. combination 3.25/1,000 person-years (HR=0.67, 95% CI 0.56- 0.81, P＜0.001)；  Secondary prevention cohort: intensity statin 19.5/1,000 person-years vs. combination 15.7/1,000 person-years (HR=0.80, 95% CI 0.70- 0.91, P＜0.001) | Liver disease: 8.6% vs. 7.8%, P＜0.05  Muscle-related adverse events: 0.7% vs. 0.5%, P＜0.05  Diabetes-related hospitalizations: 9.8% vs. 8.3%, P＜0.05 | Not reported |

SHARP: Study of Heart and Renal Protection; IMPROVE-IT: Improved Reduction of Outcomes: Vytorin Efficacy International Trial; PL-ACS: Polish Registry of Acute Coronary Syndromes; RACING: Randomized comparison of efficacy and safety of lipid-lowering with statin monotherapy versus statin/ezetimibe combination for high-risk cardiovascular diseases; ROSETTA-Stroke: Rosuvastatin combined with Ezetimibe Treatment for Target LDL-C goal Achievement in patients with recent ischemic Stroke; MESIA: Medium-intensity statin with ezetimibe versus high-intensity statin in acute ischemic cerebrovascular disease; ASCVD: Atherosclerotic Cardiovascular Disease; MI: Myocardial Infarction; ACS: Acute Coronary Syndrome; CI: Confidence Interval; RR: Relative Risk; HR: Hazard Ratio; OR: Odds Ratio; LDL-C: Low-Density Lipoprotein Cholesterol; CKD: Chronic Kidney Disease; DES: Drug-Eluting Stent; T2DM: Type 2 Diabetes Mellitus; PCI: Percutaneous Coronary Intervention; CV: Cardiovascular; HF: Heart Failure; AE: Adverse Event; MPR: Medication Possession Ratio.

**References**

Amarenco, P., Bogousslavsky, J., Callahan, A.R., Goldstein, L.B., Hennerici, M., Rudolph, A.E., et al. (2006). High-dose atorvastatin after stroke or transient ischemic attack. *N. Engl J. Med* 355, 549-559. doi: 10.1056/NEJMoa061894.

Baigent, C., Landray, M.J., Reith, C., Emberson, J., Wheeler, D.C., Tomson, C., et al. (2011). The effects of lowering LDL cholesterol with simvastatin plus ezetimibe in patients with chronic kidney disease (study of heart and renal protection): a randomised placebo-controlled trial. *Lancet* 377, 2181-2192. doi: 10.1016/S0140-6736(11)60739-3.

Cannon, C.P., Blazing, M.A., Giugliano, R.P., McCagg, A., White, J.A., Theroux, P., et al. (2015). Ezetimibe added to statin therapy after acute coronary syndromes. *N. Engl J. Med* 372, 2387-2397. doi: 10.1056/NEJMoa1410489.

Cha, J., Kim, J.H., Hong, S.J., Lim, S., Joo, H.J., Park, J.H., et al. (2025). Safety and efficacy of moderate-intensity statin with ezetimibe in elderly patients with atherosclerotic cardiovascular disease. *J. Intern Med* 297, 400-408. doi: 10.1111/joim.20029.

Choo, E.H., Moon, D., Choi, I.J., Lim, S., Lee, J., Kang, D., et al. (2024). Efficacy and diabetes risk of moderate-intensity statin plus ezetimibe versus high-intensity statin after percutaneous coronary intervention. *Cardiovasc. Diabetol.* 23, 396. doi: 10.1186/s12933-024-02498-3.

Collins, R., Armitage, J., Parish, S., Sleight, P., Peto, R. (2004). Effects of cholesterol-lowering with simvastatin on stroke and other major vascular events in 20536 people with cerebrovascular disease or other high-risk conditions. *Lancet* 363, 757-767. doi: 10.1016/S0140-6736(04)15690-0.

Hong, K., Bang, O.Y., Park, J., Jung, J., Lee, S., Song, T., et al. (2023). Moderate-intensity rosuvastatin plus ezetimibe versus high-intensity rosuvastatin for target low-density lipoprotein cholesterol goal achievement in patients with recent ischemic stroke: a randomized controlled trial. *J. Stroke* 25, 242-250. doi: 10.5853/jos.2022.02957.

Jang, J., Kim, S., Cho, J., Chun, S., You, S.C., Kim, J. (2024). Comparative effectiveness of moderate-intensity statin with ezetimibe therapy versus high-intensity statin monotherapy in patients with acute coronary syndrome: a nationwide cohort study. *Sci. Rep.* 14, 838. doi: 10.1038/s41598-024-51310-5.

Jun, J.E., Jeong, I., Ahn, K.J., Chung, H.Y., Hwang, Y. (2024). Combination of low- or moderate-intensity statin and ezetimibe vs. High-intensity statin monotherapy on primary prevention of cardiovascular disease and all-cause death: a propensity-matched nationwide cohort study. *Eur. J. Prev. Cardiol.* 31, 1205-1213. doi: 10.1093/eurjpc/zwae081.

Kim, B., Hong, S., Lee, Y., Hong, S.J., Yun, K.H., Hong, B., et al. (2022). Long-term efficacy and safety of moderate-intensity statin with ezetimibe combination therapy versus high-intensity statin monotherapy in patients with atherosclerotic cardiovascular disease (RACING): a randomised, open-label, non-inferiority trial. *Lancet* 400, 380-390. doi: 10.1016/S0140-6736(22)00916-3.

LaRosa, J.C., Grundy, S.M., Waters, D.D., Shear, C., Barter, P., Fruchart, J., et al. (2005). Intensive lipid lowering with atorvastatin in patients with stable coronary disease. *N. Engl J. Med* 352, 1425-1435. doi: 10.1056/NEJMoa050461.

Lee, S., Joo, J.H., Park, S., Kim, C., Choi, D., Hong, S., et al. (2023). Combination lipid-lowering therapy in patients undergoing percutaneous coronary intervention. *J. Am Coll Cardiol* 82, 401-410. doi: 10.1016/j.jacc.2023.05.042.

Lee, S., Joo, J.H., Park, S., Kim, C., Choi, D., Lee, Y., et al. (2025). Combination therapy with moderate-intensity atorvastatin and ezetimibe vs. High-intensity atorvastatin monotherapy in patients treated with percutaneous coronary intervention in practice: assessing RACING generalizability. *Eur. Heart J. Cardiovasc. Pharmacother.* 10, 676-685. doi: 10.1093/ehjcvp/pvad083.

Lewek, J., Niedziela, J., Desperak, P., Dyrbus, K., Osadnik, T., Jankowski, P., et al. (2023). Intensive statin therapy versus upfront combination therapy of statin and ezetimibe in patients with acute coronary syndrome: a propensity score matching analysis based on the PL-ACS data. *J. Am. Heart Assoc.* 12, e030414. doi: 10.1161/JAHA.123.030414.

Lu, Z., Kou, W., Du, B., Wu, Y., Zhao, S., Brusco, O.A., et al. (2008). Effect of xuezhikang, an extract from red yeast chinese rice, on coronary events in a chinese population with previous myocardial infarction. *Am J. Cardiol* 101, 1689-1693. doi: 10.1016/j.amjcard.2008.02.056.

Lv, X., Liu, X., Peng, Y., Li, W., Wang, J., Chen, X., et al. (2024). Medium-intensity statin with ezetimibe versus high-intensity statin in acute ischemic cerebrovascular disease (MESIA): a randomized clinical trial. *J. Stroke Cerebrovasc. Dis.* 33, 107647. doi: 10.1016/j.jstrokecerebrovasdis.2024.107647.

Park, S.Y., Jun, J.E., Jeong, I., Ahn, K.J., Chung, H.Y., Hwang, Y. (2024). Comparison of the efficacy of ezetimibe combination therapy and high-intensity statin monotherapy in type 2 diabetes. *J. Clin Endocrinol Metab* 109, 1883-1890. doi: 10.1210/clinem/dgad714.

Rouleau, J. (2005). Improved outcome after acute coronary syndromes with an intensive versus standard lipid-lowering regimen: results from the pravastatin or atorvastatin evaluation and infection therapy-thrombolysis in myocardial infarction 22 (PROVE IT-TIMI 22) trial. *Am J. Med* 118 Suppl 12A, 28-35. doi: 10.1016/j.amjmed.2005.09.014.

Schwartz, G.G., Olsson, A.G., Ezekowitz, M.D., Ganz, P., Oliver, M.F., Waters, D., et al. (2001). Effects of atorvastatin on early recurrent ischemic events in acute coronary syndromes: the MIRACL study: a randomized controlled trial. *Jama* 285, 1711-1718. doi: 10.1001/jama.285.13.1711.

Shepherd, J., Cobbe, S.M., Ford, I., Isles, C.G., Lorimer, A.R., MacFarlane, P.W., et al. (1995). Prevention of coronary heart disease with pravastatin in men with hypercholesterolemia. West of scotland coronary prevention study group. *N. Engl J. Med* 333, 1301-1307. doi: 10.1056/NEJM199511163332001.

Sohn, M., Park, Y., Lim, S. (2025). Comparative efficacy of initial statin and ezetimibe combination versus statin monotherapy on cardiovascular outcomes in diabetes mellitus: a nationwide cohort study. *Diabetes Metab. J.* doi: 10.4093/dmj.2024.0482.

Zhao, S.P., Yu, B.L., Peng, D.Q., Huo, Y. (2014). The effect of moderate-dose versus double-dose statins on patients with acute coronary syndrome in china: results of the CHILLAS trial. *Atherosclerosis* 233, 707-712. doi: 10.1016/j.atherosclerosis.2013.12.003.
